# Fiber Composition of the Grasscutter (*Thryonomys swinderianus*, Temminck 1827) Thigh Muscle: An Enzyme-histochemical Study

**DOI:** 10.4172/2157-7099.1000311

**Published:** 2015-03-01

**Authors:** Serge Niangoran Bakou, Gualbert Simon Nteme Ella, Serge Aoussi, Lydie Guiguand, Yannick Cherel, Agathe Fantodji

**Affiliations:** 1 Department of Biological Sciences and Animal Production, E.I.S.M.V. de Dakar, B.P. 5077, Senegal-Dakar fann; 2 Institut Pasteur de Côte d’Ivoire (IPCI), Senegal; 3 Department of Food Science and Engineering, Nantes-Atlantic National College of Veterinary Medicine, Nantes-France; 4 Laboratory of Animal Biology and Cytology, Abidjan 02, Côte d’Ivoire

**Keywords:** Skeletal muscle, Muscle fiber types, Histochemistry, Grasscuter

## Abstract

The aim of this study was to describe de fiber composition in the thigh muscles of grass cutter (*Thryonomys swinderianus*, Temminck 1827). Ten 4 to 6-month-old (3 to 4 kg) male grasscutter were used in this study. Eleven skeletal muscles of the thigh [M. biceps femoris (BF), M. rectus femoris (RF), M. vastus lateralis (VL), M. vastus medialis (VM), M. tensor fasciae latae (TFL), M. semitendinosus (ST), M. semimembranosus (SM), M. semimembranosus accessorius (SMA), M. Sartorius (SRT), M. pectineus (PCT), M. adductor magnus (AM)] were collected after animals euthanasia and examined by light microscopy. Three muscle fiber types (I, IIB and IIA) were found in these muscles using enzyme histochemical techniques [myosine adenosine triphosphatase (ATPase) and nicotinamide adenine dinucleotide tetrazolium reductase (NADH-TR)]. Ten of these eleven muscles are composed by 89% to 100% of fast contracting fibers (types IIA and IIB), while the SMA was almost exclusively formed by slow contracting fibers.

## Introduction

The grasscutter (*Thryonomys swinderianus*, Temminck 1827) is a hystricomorph rodent whose breeding in close captivity grows increasingly in West and Central Africa for both food speculation and wildlife resource management [1-5]. In fact, grasscutter meat is highly valued by African game consumers, whether urban or rural for its organoleptic qualities.

The meat corresponds to striated skeletal muscles of meat animals, delicatessen and game meat. Because of their contractile properties, they can engage anatomical structures to which they are attached to, and are as such active motion organs.

Structurally, vertebrate striated skeletal muscle is mainly composed of fiber muscles arranged in clusters through the connective tissue. In the conjunctiva frame we can find neurovascular structures and clusters of adipocytes that form intramuscular adipose tissue [6].

Within a given muscle, the fibers differ in their morphological, physiological and biochemical characteristics. This heterogeneity of muscle tissue reflects its high degree of specialization and is the basis of its diversity [7].

In rabbits, a phylogenetically close species to the grass cutter, there are in fact two broad categories of muscle fibers in terms of the speed of contraction: some slow (type I fibers) and others fast (fiber Type IIA, IIB and IIX) [8]. The energy required for the contraction of the muscle fibers comes from the hydrolysis of adenosine di - phosphate (ADP) catalysed by the myofibrillar ATPase. Contractile properties of muscle fibers depend on the ATPase type carried by myosin heads [9], the main protein constituent of myofibrils. The myosin molecule consists of two polymorphs of heavy chains (200 kDa), the actual carriers of the ATPase activity, to which four light chains (16-30 kDa) are associated. The speed of contraction of muscle fibers thus depends directly on their specific isoform composition of slow myosin heavy chain (type I) and fast (type IIa, IIX and IIB) [10].

To ensure their effective functioning, the muscle fibers have permanent enzymatic equipment for regenerating the hydrolyzed ATP during muscle contraction. ATP synthesis is provided by the catabolism of energy substrates such as glucose and its storage form, glycogen and fat. The stock of ATP can be reconstituted anaerobically (glycolytic) and/or aerobic (oxidative). The measurement of the activity of certain enzymes [Succinate Dehydrogenase (SDH) and Cytochrome oxidase nicotinamide adenine dinucleotide-tetrazolium reductase (NADH-TR)] involved in either of these two channels allows the assessment of their respective importance and thus to distinguish the glycolytic metabolism fibers from the oxydo-glycolytic or oxidative ones.

The purpose of this study is to present the composition type of muscle fibers of grasscutter thigh (*Thryonomys swinderianus*, Temminck, 1827) using histochemical techniques.

## Materials and Methods

### Ethics consideration

The study was approved by the Ethics Committee of Ecole Inter-Etats des Sciences et Médecine Vétérinaires (EISMV) of Dakar (Senegal).

### Animal source

Ten 4 to 6-month-old (3 to 4 kg) male grasscutters were used in this study. The animals were purchased from different grass cutter farms in the peri-urban area of Abidjan (Côte d’Ivoire). They were transferred into standard grasscutter breeding cages at the Department of Natural Sciences, Nangui Abrogua University, Abidjan, Côte d’Ivoire and fed with grass and commercial feed for a while before sacrifice. Water was given *ad libitum* during the period.

### Experimental design

Sample collection: Each animal was weighed alive and sacrificed after anaesthesia with a mixture of equal volumes of xylazin hydrochlorate (Rompun ND) and ketamine hydrochlorate (Imalgen 1000 ND), at 0.1 ml/kg of body weight.

Eleven muscles [M. biceps femoris (BF), M. rectus femoris (RF), M. vastus lateralis (VL), M. vastus medialis (VM), M. tensor fasciae latae (TFL), M. semitendinosus (ST), M. semimembranosus (SM), M. semimembranosus accessorius (SMA), M. sartorius (SRT), M. pectineus (PCT), M. adductor magnus (AM)] of the thigh were removed 15 minutes after the sacrifice (Table 1).

### Histochemical processing

A transverse section (about 0.5 cm) was obtained from the muscle midbelly and frozen for 30 s at −80°C in isopentane previously cooled with liquid nitrogen. Sections at 12 μm were cut using a cryostat and then treated with histochemical techniques. Nicotinamide adenine tetrazolium-reductase (NADH-TR) reaction according to Dubowitz [11] to characterise fiber metabolism and myofibrillar, adenosine triphosphatse (ATPase) reaction after acid pre-incubation (pH 4.35 and 4.6) and alkaline pre-incubation (pH 10.4) according to Guth and Samaha [9] to detect ATPase activity within fibers.

### Morphometric analysis

The different types of fibers were determined according to the classification of Brooke and Kaiser [12].

Morphometric parameter was assessed using a semi-automatic image analysis system (NIS – Elements AR 3,1 image analyser, Nikon Laboratory Imaging, Prague, Czech Republic). Thus, the relative proportion of the different fibers types (I, IIA and IIB) in thigh muscles was determined from an average count of 300 fibers in 6 random fields. Determination of the proportion of different muscle fiber types were performed on sections of frozen muscles treated with acid ATPase (pH 4.35) [13-15].

### Statistical analysis

Conventional statistical procedures have been used to calculate mean and standard deviation (S.D.). The coefficient of variation for observational bias (repeatability) was about 3%.

## Results

### Morphology

**Figure 1:**
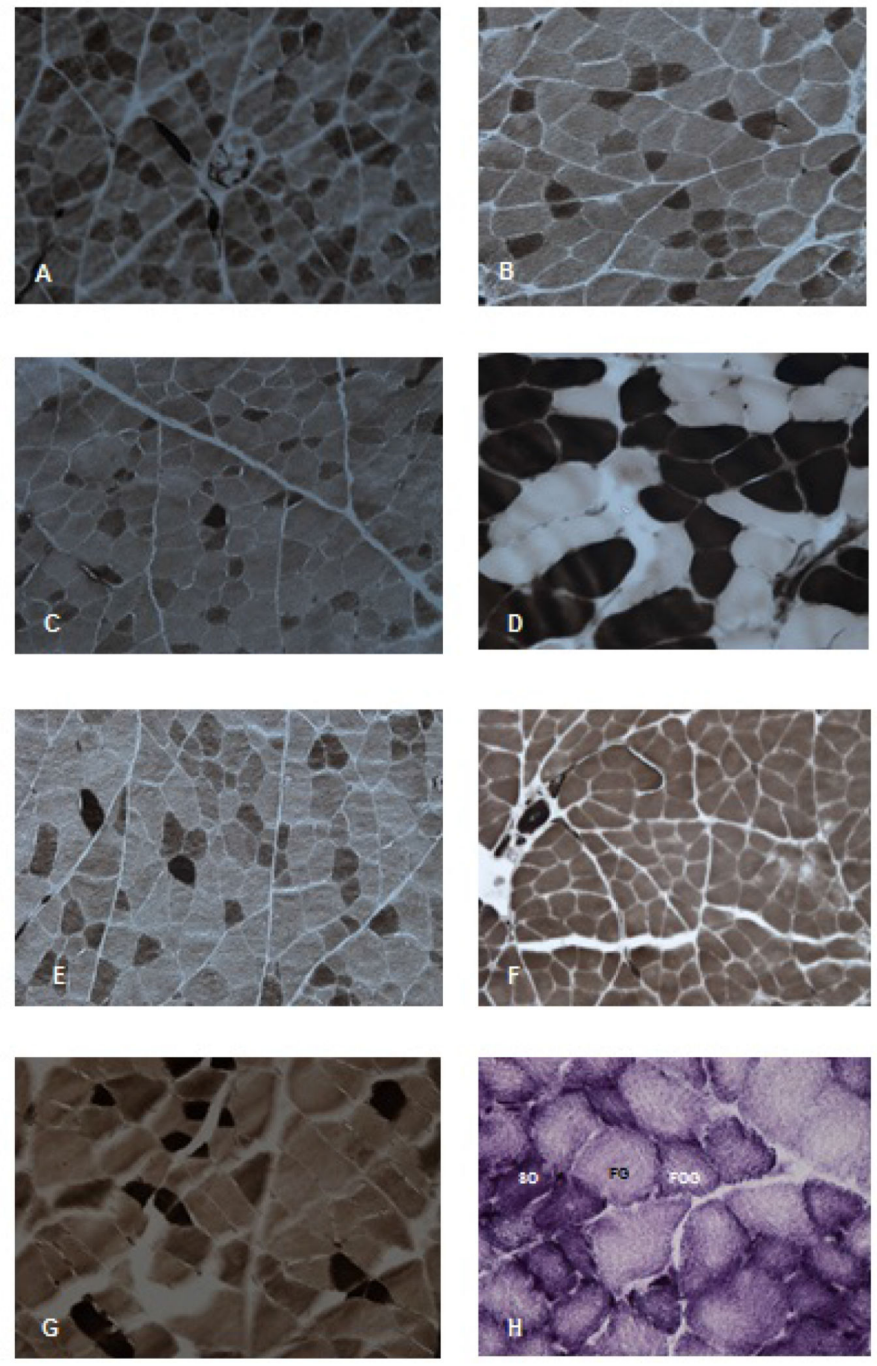
Light photomicrographs demonstrating the features of different muscles use in our study. Cross-sections from rectus femoris (A), Sartorius (B), biceps femoris (C), vastus medialis (D), adductor magnus (E), semimembranosus accessorius (F), pectineus (G) muscles stained by myofibrillar ATPase after acid preincubation (pH 4.2). These muscles are mixed and composed of type I (dark), type IIB (light) and type IIA (intermediate) fibres. Cross section from vastus lateralis muscle stained by NADH-Tetrazolium reductase (SO: Slow oxidative fibres; FOG: Fast oxydo-glycolytic fibres; FG: fast glycolytic fibres).

Most of the muscles studied were characterized by a mosaic pattern of polygonally shaped fibers with the exception of M. semimembranosus accesorius, which exhibited an exclusively slow-twitch fibers pattern. We found no evidence of fiber type grouping or marked regionalisation of distinct fiber types in all the other muscles studied (Figure 1).

### Fiber type

Data on the composition of the fiber type in the muscles of the grasscutter thigh are shown in Table 1.

The reaction to the myofibrillar ATPase acid (pH 4.35) revealed three types of fibers (I, IIA and IIB) in most of the muscles of the grasscutter thigh (Figure 1). Thus, in all muscles examined, M. semimembranosus accesorius is the only homogeneous fiber containing only type I fibers. Mm. rectus femoris and sartorius are almost exclusively composed of type IIB fibers. The other muscles of the grasscutter thigh (M. adductor magnus, M. biceps femoris, M. pectineus, M. semimembranosus, M. semitendinosus, M. tensor fascia latae, M. vastus lateralis and M. vastus medialis) are heterogeneous composed of the three fiber types I, IIA and IIB.

In heterogeneous muscles type IIB fibers are the most numerous (76.48%a to 99%a). Type IIA fibers come in second place and represent 18.88%, 8.58% and 8.04% in Mm adductor magnus, biceps femoris and vastus lateralis respectively. Finally, type I fibers are fewer in all heterogeneous muscles of the grasscutter thigh except for M. pectineus where they account for 11.78% of total fibers.

## Discussion

In general, the muscles of grasscutter thigh, has a fiber composition similar to that of the pelvic limb of other rodents in particular the guinea pig (*Cavia porcellus*) and rat (*Rattus rattus*) and the rabbit (*Oryctolagus cuniculus*) a lagomorph which is a phylogenetically close species [10,15-21]. Indeed, the reaction to the myofibrillar ATPase revealed the existence of three types of fibers which according to the classification of Brooke and Kaiser [12] correspond to fiber types I, IIA and IIB. However, the distribution of these three types of muscle fibers varies from one to another and enables to distinguish three main types of muscles in the grasscutter thigh: (i) a homogeneous muscle exclusively composed of type I fibers (SMA), (ii) almost exclusively composed of type IIB fibers (SM and RF) muscles, (iii) and heterogeneous muscles composed of fibers type I, IIA and IIB (AM, BF, PCT, SM, ST, TFL, VL and VM).

Except for M. adductor magnus, the results of this study clearly show that the muscles of grasscutter thigh are mostly composed of rapid and glycolytic fibers (type IIB). For example, M. rectus femoris, M. Sartorius, M. vastus medialis, M. semimembranosus and M. tensor fascia latae are composed of fast glycolytic fibers with an aspect ratio of 99 ± 1%, 98.1 ± 1.43%, 97.5 ± 1.97%, 96.64 ± 2.17% and 94.02 ± 2.19% respectively. This high proportion of fast glycolytic fibers in the thigh muscles of the grasscutter is consistent with the observations reported by some authors in the rabbit [8,15]. Furthermore, it is well established that the presence of this high proportion of type IIB fibers correlates with the means of locomotion of the rabbit, a species that moves using successive leaps because of the greater length of its hind limbs compared to its fore limbs. The grasscutter is a digitigrade rodent that is nonetheless capable of making spectacular leaps [22]. Also, type IIB fibers that make up his thigh muscles could explain this ability of this species. Among the thigh muscles studied, M. semimembranosus accessorius is entirely composed of oxidative slow-twitch fibers (type I). The M. semimembranosus accessorius in the grasscutter is a fusiform muscle housed within the M. semimembranosus with which it shares the same origin (ischiatic tuberosity) but ends on the medial side of the distal end of the femur [23]. M. semimembranosus unlike M. semimembranosus accessorius contains very little type I fibers (1.22 ± 1.14%). Our results are opposed to those reported by Bacou et al. [17] in rabbits. Indeed, these authors reported that M. semimembranosus proprius contains 100% of oxidative slow-twitch fibers (type I), while it’s dependent, the M. semimembranosus accessorius is composed of fast glycolytic fibers (type IIB) and oxidative - glycolytic (type IIA). Although the reaction to the myofibrillar ATPase is widely used for typing of muscle fibers, this method does not always manage to highlight all type II fibers [24]. This is why the use of immunohistochemistry, which the benefit lies in the fact that the isoforms may be detected by appropriate antibody, regardless of their phenotypic characteristics must complete this last method for a better typing of muscle fiber [25].

Different types observed in the thigh muscles of the grasscutter are widely represented both in mammals and in birds [13,25]. They reflect the phenomenon of “fibers regionalization” described for the first time by Gordon and Phillips in 1955 [26]. According to these authors, the distribution of fibers in skeletal muscles of mammals is “regionalized” with slow fibers located in deep region near the bone and fast fibers at the surface area (subcutaneous). This assertion is consistent with our results. Indeed, the deep muscles of the thigh as Mm. semimembranosus accessorius, pectineus, and adductor magnus are those that contain the most slow-twitch oxidative ie respectively, 100%, 11.78 ± 3.27% and 4.64 ± 2.17%. Mechanisms and functional implications of the phenomenon of regionalization remain to this day not very well known. These mechanisms seem to take place during the embryonic development of muscle fibers [27-29]. Thus, in the muscles of the pelvic limb in rats [28], cattle [27] pig and guinea pigs [29], the first generation of myotubes give rise to slow type primary fibers. They provide a subsequent frame for the formation of a second generation of myotubes, a larger number that will give fast type secondary fibers. This arrangement in concentric bundles slow and fast fibers persists during the postnatal growth of the pig [29]. However, during fetal development and during part of the postnatal growth, some secondary fibers (fast) change type and become slow and other new bundles of slow fibers appear. In addition, Condon et al. [28], explains the phenomenon of regionalization by the presence of “fast or slow gradients morphogenetic” that result in the transition from slow to fast isoform of the myosin and vice versa.

Moreover, according to Brandstetter et al. [27], innervation and muscular work influence the regionalization of fiber types acquired during embryonic life in adult muscles. Many works carried out on the neuromuscular system, studied the interactions between muscle and motor nerve and tried to explain the specificity of the association between neurons and fibers of the same type [30]. In this work, two different but non-exclusive hypotheses have been advanced. The first is that the characteristics of the muscle fibers are genetically different and neurons recognize each type of fiber, the second is that the fibers are genetically equipotential and their specificity is determined by the nature of the neuron that innervates them [31].

Adult skeletal striated muscles have no autonomous activity and they cannot be dissociated from motor nerves that control their activity. They form with the motoneuron, which innervates them the drive unit representing the functional entity of the muscular contraction.

According to the theory of Henneman [32], motor units formed by oxidative slow fibers are recruited for movements that require a slow speed and low contraction force (standing, walking). Motor units formed by the fast oxidative-glycolytic fibers are recruited for movements that require high speed and high force contraction (race). Finally, the motor units formed by fast glycolytic fibers are recruited for fast and powerful movements (jumping). Thus, the balance of glycolytic fibers in Mm. rectus femoris, sartorius, vastus medialis and semimembranosus observed in our study, have a functional explanation. Furthermore, in mammalian quadrupeds, chest members which are closer to the centre of gravity of the animal are mostly sustainers, while the pelvic limbs are mostly thrusters. The muscles of the thoracic limb will therefore be involved in the coordination of body balance during the execution of a movement. For this reason, they are composed by motor units formed mainly by oxidative fibers. However, the muscles of the pelvic limb involved in all movements requiring great strength (jumping, rearing, startup, race) consist of drive units formed mostly by glycolytic fibers.

In conclusion, the type of fiber composition of the grasscutter thigh muscles is directly related to the functions of the pelvic limb region muscles.

## Figures and Tables

**Table 1:** Proportion (means ± S.D.) of type I, type IIA and type IIB, expressed as a percentage of 300 fibres counted in thigh muscle of grass cutter (*Thryonomys swinderianus*, Temminck 1827).

Muscle	n	Type I	Type IIA	Type IIB
AM	10	4.64 ± 0.99	18.88 ± 6.20	76.48 ± 5.37
BF	10	2.90 ± 1.35	8.58 ± 1.18	88.52 ± 0.52
PCT	10	11.78 ± 3.27	3.72 ± 0.46	84.50 ± 3.57
RF	10	1.00 ± 1.00	0.00 ± 0.00	99.00 ± 1.00
SRT	10	1.90 ± 1.43	0.00 ± 0.00	98.10 ± 1.43
SM	10	1.22 ± 1.14	2.14 ± 1.62	96.64 ± 2.17
SMA	10	100 ± 0.00	0.00 ± 0.00	0.00 ± 0.00
ST	10	1.62 ± 0.61	4.68 ± 0.63	93.70 ± 0.70
TFL	10	1.82 ± 1.31	4.16 ± 1.41	94.02 ± 2.19
VL	10	5.24 ± 1.78	8.04 ± 3.26	86.72 ± 3.33
VM	10	1.42 ± 1.41	1.08 ± 0.72	97.50 ± 1.97

## References

[1] Asibey EOA (1973). The grass cutter, Thryonomys swinderianus Temminck, in Ghana. Symp Zool Lond.

[2] Ajayi SS, Tewe O (1980). Food preference and carcass composition of the grass cutter (thryonomys swinderianus) in captivity. Afr J Ecol.

[3] Alogninouwa TH, Yewadan LT, Schrage R (1993). L’aulacode, une espèce africaine intéressante à plus d’un titre. Bull. Mens. Office National de la Chasse.

[4] Jori F, Mensah GA, Adjanohoun E (1995). Grasscutter farming: an example of rational utilization of wildlife. Biodiversity and Conservation.

[5] Edderai D, Ntsame M, Houben P (2001). Gestion de la reproduction en aulacodiculture. synthèse des outils et méthodes existants. INRA Prod Anim.

[6] Gondret F, Bonneau M (1998). Mise en place des caractéristiques du muscle chez le lapin et incidence sur la qualité de la viande. INRA Prod Anim.

[7] Pette D, Staron RS (1990). Cellular and molecular diversities of mammalian skeletal muscle fibers. Rev Physiol Biochem Pharmacol.

[8] Hämäläinen N, Pette D (1993). The histochemical profiles of fast fiber types IIB, IID, and IIA in skeletal muscles of mouse, rat, and rabbit. J Histochem Cytochem.

[9] Guth L, Samaha FJ (1972). Erroneous interpretations which may result from application of the “myofibrillar ATPase” histochemical procedure to developing muscle. Exp Neurol.

[10] Peter JB, Barnard RJ, Edgerton VR, Gillespie CA, Stempel KE (1972). Metabolic profiles of three fiber types of skeletal muscle in guinea pigs and rabbits. Biochemistry.

[11] Dubowitz V (1985). Muscle biopsy: A practical approach.

[12] Brooke MH, Kaiser KK (1970). Muscle fiber types: how many and what kind?. Arch Neurol.

[13] Bakou S, Cherel Y, Gabinaud B, Guigand L, Wyers M (1996). Type-specific changes in fibre size and satellite cell activation following muscle denervation in two strain of turkey (Meleagris gallopavo). J Anat.

[14] Kostrominova TY, Reiner DS, Haas RH, Ingermanson R, McDonough PM (2013). Automated methods for the analysis of skeletal muscle fiber size and metabolic type. Int Rev Cell Mol Biol.

[15] Rab M, Neumayer Ch, Koller R, Kamolz L-P, Haslik W (2000). Histomorphology of rabbit thigh muscles: establishment of standard control values. J Anat.

[16] Alnaqeeb MA, Goldspink G (1987). Changes in fibre type, number and diameter in developing and ageing skeletal muscle. J Anat.

[17] Ariano MA, Armstrong RB, Edgerton VR (1973). Hindlimb muscle fiber populations of five mammals. J Histochem Cytochem.

[18] Bacou F, Rouanet P, Barjot C, Janmot C, Vigneron P (1996). Expression of myosin isoforms in denervated, cross-reinnervated, and electrically stimulated rabbit muscles. Eur J Biochem.

[19] Delmas D, Ouhayoun J (1990). Technologie de l’abattage du lapin. 1. Etude descriptive de la musculature. Viandes Produits Carnés.

[20] Ouali A, Dufour E, Obled A, Deval C, Valin C (1987). Action des protéinases musculaires sur les myosines rapide et lente. Relation avec la protéolyse post mortem dans les muscles de type contractile variable. Reprod Nutr Dev.

[21] Maltin CA, Delday MI, Baillie AG, Grubb DA, Garlick PJ (1989). Fiber-type composition of nine rat muscles. I. Changes during the first year of life. Am J Physiol.

[22] Adjanohoun E (1992). Le cycle sexuel et la reproduction de l’aulacode (Thryonomys swinderianus – Temminck, 1827). Mammalia.

[23] Nteme-Ella GS, Aoussi S, Bakou SN, Ouassat M, Costiou P (2010). Etude descriptive des muscles du grand aulacode (Thryonomys swinderianus, Temminck 1827). RASPA.

[24] Dahl HA, Roald L (1991). How unequivocal is the muscle fibre type concept?. Anat Embryol (Berl).

[25] Rowlerson A (1994). An outline of fibre types in vertebrate skeletal muscle: Histochemical identification and myosin isoforms. Basic Appl Myol.

[26] Kernell D (1998). Muscle regionalization. Can J Appl Physiol.

[27] Brandstetter AM, Picard B, Geay Y (1997). Regional variations of muscle fibre characteristic in m. semitendinosus of growing cattle. J Muscle Res Cell Motil.

[28] Condon K, Silberstein L, Blau HM, Thompson WJ (1990). Differentiation of muscle fibre types in the prenatal rat hind limb. Dev Biol.

[29] Stickland NC (1996). Rôles de la génétique et de l’environnement dans la variabilité du développement musculaire chez le porc et le cobaye. INRA Prod Anim.

[30] McArdle JJ (1983). Molecular aspects of the trophic influence of nerve on muscle. Prog Neurobiol.

[31] Bacou F, Vigneron P (1988). [Properties of skeletal muscle fibers. Influence of motor innervation]. Reprod Nutr Dev.

[32] Henneman E, Clamann HP, Gillies JD, Skinner RD (1974). Rank order of motoneurons within a pool: law of combination. J Neurophysiol.

